# The C-terminal region of *Bfl-1 *sensitizes non-small cell lung cancer to gemcitabine-induced apoptosis by suppressing *NF-κB *activity and down-regulating Bfl-1

**DOI:** 10.1186/1476-4598-10-98

**Published:** 2011-08-16

**Authors:** Min-Kyoung Kim, Yoon-Kyung Jeon, Jong-Kyu Woo, Yun Choi, Dae-Han Choi, Yeul-Hong Kim, Chul-Woo Kim

**Affiliations:** 1 Department of Pathology, Cancer Research Institute, Seoul National University College of Medicine, 28 Yeongeon-dong, Jongno-gu, Seoul 110-799, South Korea; 2 Tumor Immunity Medical Research Center, Cancer Research Institute, Seoul National University College of Medicine, 28 Yeongeon-dong, Jongno-gu, Seoul 110-799, South Korea; 3 Department of Internal Medicine, Korea University College of Medicine, Anam-dong 5-Ga, Seongbuk-gu, Seoul 136-705, South Korea

**Keywords:** gemcitabine, NF-κB, Bfl-1, gene therapy, non-small cell lung cancer

## Abstract

Gemcitabine is used to treat several cancers including lung cancer. However, tumor cells often escape gemcitabine-induced cell death via various mechanisms, which include modulating bcl-2 family members and NF-κB activation. We previously reported that the C-terminal region of Bfl-1 fused with GFP (BC) is sufficient to induce apoptosis in 293T cells. In the present study, we investigated the anti-tumor effect of combined BC gene therapy and gemcitabine chemotherapy *in vitro *and *in vivo *using non-small cell lung cancer cell lines and a xenograft model. Cell lines were resistant to low dose gemcitabine (4-40 ng/ml), which induced NF-κB activation and concomitant up-regulation of Bfl-1 (an NF-κB-regulated anti-apoptotic protein). BC induced the apoptosis of A549 and H157 cells with caspase-3 activation. Furthermore, co-treatment with BC and low dose gemcitabine synergistically and efficiently induced mitochondria-mediated apoptosis in these cells. When administered alone or with low dose gemcitabine, BC suppressed NF-κB activity, inhibited the nuclear translocation of p65/relA, and down-regulated Bfl-1 expression. Furthermore, direct suppression of Bfl-1 by RNA interference sensitized cells to gemcitabine-induced cell death, suggesting that Bfl-1 importantly regulates lung cancer cell sensitivity to gemcitabine. BC and gemcitabine co-treatment also showed a strong anti-tumor effect in a nude mouse/A549 xenograft model. These results suggest that lung cancer cells become resistant to gemcitabine via NF-κB activation and the subsequent overexpression of Bfl-1, and that BC, which has both pro-apoptotic and NF-κB inhibitory effects, could be harnessed as a gene therapy to complement gemcitabine chemotherapy in non-small cell lung cancer.

## Introduction

Lung cancer remains a leading cause of cancer-related death [1] despite the introduction of several types of cytotoxic agents. In non-small cell lung cancer (NSCLC), chemotherapy often achieves limited clinical improvements due to acquired drug resistance and intolerable toxicities. Gemcitabine (difluorodeoxycytidine hydrochloride, dFdC) is a deoxycytidine analogue that is converted *in vivo *into the active metabolites, difluorodeoxycytidine di- and triphosphate (dFdCDP, dFdCTP). DFdCDP acts by inhibiting ribonucleotide reductase, whereas dFdCTP is incorporated into DNA and prevents DNA synthesis, thereby inducing apoptosis. Gemcitabine has been approved by the Food and Drug Administration (FDA) as a treatment for advanced and metastatic pancreatic cancer, ovarian cancer, breast cancer, and NSCLC, alone or in combination with other drugs http://www.cancer.gov/cancertopics/druginfo/gemcitabinehydrochloride. Clinical trials have demonstrated that gemcitabine prolongs survival and improves the quality of life of advanced NSCLC patients [2]. In fact, gemcitabine is considered to be one of the most effective agents for treating NSCLC. Previous studies have concluded that when used as a single agent, gemcitabine consistently yields response rates exceeding 20%. Furthermore, preclinical data indicate that when used with platinum compounds, such as, cisplatin or carboplatin, gemcitabine has synergistic anti-tumor effects [3,4].

However, gemcitabine often fails to achieve adequate disease control due to intrinsic or acquired resistance of tumor cells. The following are representative examples of putative resistance mechanisms; NF-κB and PI3K/Akt pathway activation in pancreatic and breast cancer [5,6] the up-regulation of anti-apoptotic Bcl-2 protein in pancreatic cancer [7,8] the deficiency of human equilibrate nucleoside transporter 1 in NSCLC [9]and alterations of gemcitabine metabolizing enzymes [10-13]. Many of these chemo-resistant mechanisms involve interrupting the apoptotic pathway [14]. In particular, increased expression of anti-apoptotic bcl-2 family proteins stabilizes the mitochondrial membrane, and thus, elevates the apoptotic threshold. Accordingly, the suppression of Bcl-2 using siRNA (small interfering RNA) restores gemcitabine sensitivity in pancreatic cancer cells [8]. The anti-apoptotic effects of chemotherapy-induced NF-κB activation are mediated by a series of molecules regulated by NF-κB, and by inhibiting NF-κB tumor cells can be sensitized to chemotherapeutic agents, such as, gemcitabine [15-17]. For example, the inhibition of NF-κB by bortezomib (a proteasome inhibitor) sensitized NSCLC to gemcitabine-induced apoptosis, and the silencing of the NF-κB p65/relA subunit with siRNA increased the effectiveness of gemcitabine in a subset of pancreatic cancer cells [18,19]. In addition, resistance to gemcitabine may be overcome by using in combination with cytotoxic agents with different modes of action [7]. Of note, combined gene therapy has been proposed as a means of overcoming resistance to chemotherapy, as demonstrated by enhanced chemosensitivity via the targeted down-regulation of anti-apoptotic Bcl-2 and Bcl-x_L _[12,20-22]

Recently, Bfl-1 was found to be a direct transcriptional target of NF-κB [23-25]. Bfl-1 interacts and sequestrates the pro-apoptotic molecules, Bid and Bim, and thereby suppresses apoptosis and inhibits cytochrome c release from mitochondria [26,27]. We previously observed high levels of Bfl-1 mRNA in blood, lung, and stomach cancer cell lines [28], and separately reported that a Bfl-1/green fluorescent protein (GFP) adduct induces caspase activation and disrupts mitochondrial permeability in 293T cells. Furthermore, the domain responsible for this apoptotic function of Bfl-1 fused with GFP was mapped to 20 amino acids in its C-terminal region [17]. Therefore, we hypothesized that gene therapy based on the expression of a protein containing the C-terminal of Bfl-1 and GFP (BC) might sensitize cancer cells to gemcitabine.

In the present study, we demonstrate that low dose gemcitabine activates NF-κB and subsequently up-regulates Bfl-1 in NSCLC cells, which has been suggested to be a major mechanism of resistance to gemcitabine. We found that BC gene therapy inhibited NF-κB activity and decreased Bfl-1 expression, and restored sensitivity to gemcitabine. Furthermore, combined BC and gemcitabine therapy effectively suppressed tumor growth in a xenograft model. Accordingly, our findings suggest that combined BC gene therapy and gemcitabine chemotherapy offers a potential means of overcoming resistance to gemcitabine chemotherapy in NSCLC.

## Materials and methods

### Cell lines and reagent

HEK 293 (human embryonic kidney fibroblasts, ATCC CRL-1573™, passage-13) was grown in DMEM supplemented with 10% FBS (Invitrogen, Groningen, The Netherlands). Human NSCLC cell lines, A549 (adenocarcinoma, ATCC CCL-185, passage-23), H460 (large-cell carcinoma, ATCC HTB-177, passage-7), H157 (squamous cell carcinoma, ATCC CRL-5802, passage-18) and PC-9 (adenocarcinoma, was kindly provided by Prof. Tae-You Kim, Seoul National University College of Medicine, Seoul, Korea) were maintained in RPMI1640 containing 10% FBS. All cells were verified by morphology, growth curve analysis and tested for *Mycoplasma*. All cell lines except for PC-9 were obtained from and characterized by the American Type Culture Collection (ATCC). No further authentication was done by the authors. Gemcitabine was purchased from Eli Lilly (Suresnes, France).

### Recombinant adenovirus with Tet-off system

The cDNA fragments of human Bfl-1 corresponding to amino acids 147-175 and mouse Bax were amplified by PCR and subcloned into pEGFPC1 vector (Clontech, Palo Alto, CA) to generate GFP-Bfl-1-C-terminal (BC) and GFP-Bax, respectively, as previously described [17,29]. Bfl-1-C-terminal was fused with multi-cloning site of pEGFPC1 (not involve GFP) for external charged residues, and subcloned into pcDNA vector to generated pMBC. GFP- Bfl-1-C-terminal (BC), GFP, and Bfl-1 were subcloned into the NheI/SalI site of pTRE shuttle vector (Clontech) to generate pTRE-BC, pTRE-GFP, and pTRE-Bfl-1. GFP-Bax (gBax) was subcloned into the BglII/HindIII site of pTRE-GFP shuttle vector to generate pTRE-gBax. The pTRE-BC, -gBax, Bfl-1 and -GFP constructs were linearized by PI-SceI and I-CeuI digestion, inserted into adenoviral vector pAdenoX (Clontech), digested with PacI, and then transfected into 293 cells to generate GFP, BC, Bfl-1 and Bax-viruses. Ten days after transfection, when the cytopathic effect became evident, clear culture supernatants were obtained, and viruses were then propagated in 293 cells and purified by standard methods [30].

We used the Tet-off system to regulate gene expression, which responds equally well to either tetracycline or doxycycline [31,32]. Briefly, both BC and Tet-off adenovirus were simultaneously infected into cells, in which BC expression was inhibited in the presence of doxycycline, but induced in its absence. The efficiency of the Tet-off system was tested by examining GFP expression and cell viability. Cells were cultured in 24-well plates at a density of 1 × 10^5 ^per well. 24 h after plating, variable concentrations of Tet-off and control or BC viruses were added. Immediately after infection, doxycycline (Sigma-Aldrich, St Louis, MO) was added to a final concentration of 1 mg/ml. Multiplicity of infection (MOI) was determined by measuring the absorbance of dissociated viruses at 260 nm; one absorbance was arbitrarily set at 10^12 ^viral particles per milliliter. The particle-to-infectious unit ratio was 100:1.

### Cell viability, apoptosis, and cytochrome C release assays

The viabilities of treated cells were measured using a Cell Counting Kit (CCK)-8 (Dojindo Molecular Technologies, Kumamoto, Japan). All assays were performed in triplicate. For subG1 analysis, cells were harvested, washed, and fixed overnight with 70% ethanol. Cell pellets were re-suspended in staining buffer (10 μg/mL propidium iodide and 0.2 mg/mL RNase A in PBS), and analyzed by flow cytometry (Epics XL; Coulter, Marseille, France). Apoptotic cell death was determined by Annexin V and/or PI staining followed by flow cytometry using Annexin V-FITC (or PE) kits (BD Pharmingen, San Diego, CA). For DNA fragmentation analysis, genomic DNA was extracted, subjected to electrophoresis in 2% agarose gel, and visualized by ethidium bromide staining.

To measure cytochrome C release from mitochondria, cells were harvested and fixed with 2% paraformaldehyde for 10 min at 37°C and then permeabilized with buffer (1% BSA, 0.1% saponine, 0.1% sodium azide in PBS) for 1 h on ice. Cells were stained with anti-cytochrome C antibody (clone 6H2.B4) (BD Pharmingen) and then with PE-labeled secondary antibody (Silenus, Hawthorn, Australia), and analyzed by flow cytometry [33].

### Caspase activity assay

Cells were harvested, and then centrifuged at 450 g for 10 min at 4°C. After removing supernatant, cell pellets were resuspended in 100 μl of lysis buffer (50 mM HEPES pH 7.5, 1 mM DTT, 0.1 mM EDTA, 0.1% CHAPS), and lysed by repeated freezing at -70°C and thawing on ice. Cell lysates were cleared by centrifugation at 15,000 g for 20 min at 4°C; the resulting supernatants are referred to as cell extracts. Typically, 100 μg of cell extract was added to caspase activity assay buffer (100 mM HEPES pH 7.5, 10% sucrose, 0.1% CHAPS, 10 mM DTT, 200 μM DEVD-pNA) with or without 100 μM Boc-D. These mixtures were then incubated at 37°C for 4 h and the yellowish color caused by the release of pNA was quantified using an ELISA reader at 405 nm. Active caspase-3 levels were quantified using active caspase-3-PE staining kits (BD Pharmingen) by flow cytometry.

### Immunoblotting

Cells were harvested and centrifuged at 500 g for 10 min at 4°C. The cell pellets were lysed with 2× sample buffer (20 mM Tris pH 8.0, 2 mM EDTA, 2% SDS, 20 mM DTT, 1 mM Na3VO4, 20% glycerol). Cell lysates were boiled for 3 min, and suspensions were centrifuged at 10,000 g for 10 min at 4°C. The supernatants obtained are referred to as whole cell lysates. Typically, 50 μg of total cellular proteins were separated by SDS-PAGE and then transferred to a nitrocellulose membrane, which was subsequently subjected to immunoblotting using the following antibodies; rabbit anti-Bcl-2, -Bcl-x_L_, -Bax, -NF-κB p65/relA, and -lamin B (Santa Cruz Biotechnology, Santa Cruz, CA), and mouse anti-α-tubulin (Calbiochem, San Diego, CA). Rabbit polyclonal antibody against human Bfl-1 was produced by a commercial antibody production service (Youngin Frontier, Seoul, Korea).

### Reverse transcription-PCR (RT-PCR)

Total RNA was isolated from cells using TRIzol reagent (Invitrogen). 5 μg aliquots of total RNA were subjected to reverse transcription using a RT-PCR kit (Promega, Madison, WI). cDNAs were amplified using primers as follows; Bfl-1, forward 5'-GCTCAAGACTTTGCTCTCCACC-3' and reverse 5'-TGGAGTGTCCTTTCTGGTCAACAG-3'; Bcl-x_L_, forward 5'-GGATGGCCACTTACCTGA-3' and reverse 5'-CGGTTGAAGCGTTCCTG-3'; Bcl-2, forward 5'-GTGTGGAGAGCGTCAACC-3' and reverse 5'-GCTGGGGCCGTACAGTT-3'; β-actin, forward 5'-GGAAATCGTGCGTGACATTAAGG-3' and reverse 5'-GGCTTTTAGGATGGCAAGGGAC-3'.

### NF-κB activity assessment

NF-κB activity was assessed by detecting the nuclear translocation of NF-κB subunit or luciferase assay. To prepare nuclear extracts, cells were washed in ice cold PBS and resuspended in Buffer A (10 mM HEPES (pH7.9), 1.5 mM MgCl2, 10 mM KCl, 0.1 mM EDTA, 1 mM DTT, 1 mM PMSF, 0.6% Nonidet P-40). After incubation for 20 min on ice, nuclear pellets were recovered by centrifugation at 1200 g, and suspended in Buffer B (20 mM HEPES, 0.4 M NaCl, 1 mM EDTA, 1 mM DTT, 25% glycerol). Aliquots were then incubated at 4°C for 30 min and supernatants containing nuclear proteins were collected by centrifugation at 21000 g. The presence of NF-κB subunits in nuclear and cytosolic fractions was examined by immunoblotting using anti-p64/RelA antibodies.

For luciferase assay, cells were transiently transfected with pNF-κB-Luc or pβ-gal-lacZ plasmids using Lipofectamine 2000 (Invitrogen, Carlsbad, CA). Luciferase and β-galactosidase activities were measured using an Orion luminometer (Berthold Detection Systems. Oak Ridge, TN), and cellular protein contents were determined using the bicinchoninic acid technique (Pierce Biotechnology Inc, Rockford, IL). Luciferase activities were normalized versus lysate protein contents and β-galactosidase activity used as an internal control to determine transduction efficiency.

### RNA interference

Three types of siRNAs were synthesized by Bioneer (Daejon, Korea) to silence Bfl-1, as follows: siRNA1, sense 5'-AAGGAGUUUGAAGACGGCAUC-3' and anti-sense 3'-GAUGCCGUCUUCAAACUCCUU-5' [25]; siRNA2, sense 5'-CCUAAAUCUGGCUGGAUGACU-3' and anti-sense 3'-AGUCAUCCAGCCAGAUUUAGG-5'; siRNA3, sense 5'-GCUAUCUCUCCUGAAGCAAUACUGUUGA-3' and anti-sense 3'-UCAACAGUAUUGCUUCAGGAGAGAUAGC-5'. The sequences of the scrambled siRNAs used were sense: 5'-AAGGUCACAGAUAGAUUGGC-3' and anti-sense 3'-GCCAAUCUAUCUGUGACCTT-3'. Transfection was performed in 6-well plates at 70% confluence at a final siRNA concentration of 10 nM over 72 h using Lipofectamine 2000 (Invitrogen).

### Animal experiments

To evaluate the *in vivo *anti-tumor effect of combined BC and low dose gemcitabine therapy, we established a tumor xenograft model in nude mice. Nude mice (6 to 8-week-old; Charles River Japan Inc., Yokohama) were subcutaneously injected in the flank with 2×10^6 ^A549 cells in 100 μl PBS. When tumor volumes reached 5 to 10 mm in diameter, mice were randomly divided into four groups with 5 animals per group. Tumor-bearing mice were injected intratumorally with control (GFP) or BC adenovirus and Tet-off-adenovirus at the same concentrations (5×10^8 ^PFU in 50 μl of PBS), and intraperitoneally administered with PBS or 10 mg/kg gemcitabine. In brief, four experimental groups were treated as follows: group 1, with control adenovirus and PBS; group 2, control adenovirus and gemcitabine; group 3, BC adenovirus and PBS; group 4, BC adenovirus and gemcitabine. These co-treatments were performed three times with three days intervals. Tumor growths were evaluated twice or three times weekly for 4 weeks after the last treatment by measuring the length and width of each tumor using a caliper. Tumor volumes were calculated using m_1_^2 ^× m_2 _× 0.5236 (where m_1 _and m_2 _are the short and long tumor axes). Tumor weights were obtained using an analytical balance.

Experiments and maintenance of mice were performed according to the animal experiment guidelines at the Center for Animal Resource Development (ethical committee), Seoul National University College of Medicine.

### Statistical analysis

Data were analyzed using the Student's *t *test, and *p *values less than 0.05 were considered to be statistically significant.

## Results

### Low dose gemcitabine treatment leads to NF-κB activation and Bfl-1 up-regulation in lung cancer cells

**Figure 1:**
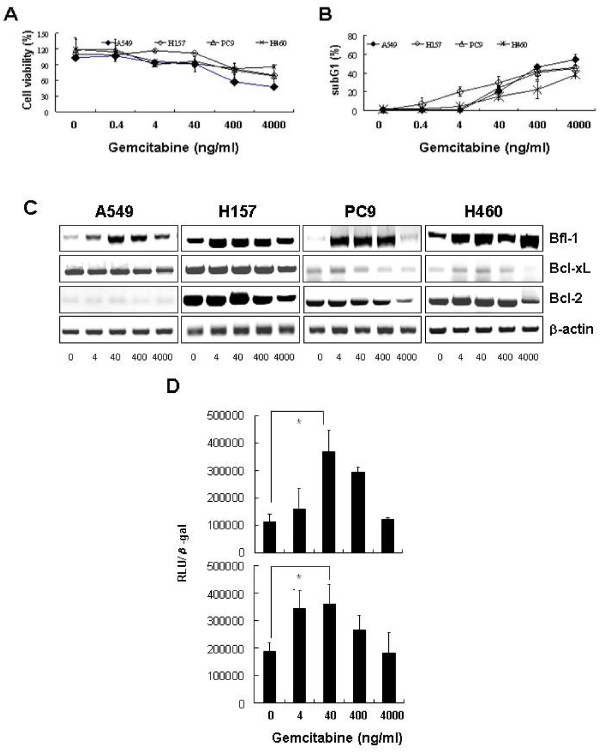
**Low dose gemcitabine cannot efficiently induce cell death in lung cancer cells, and leads to NF-κB activation and Bfl-1 overexpression**. ***A *and *B***, A549, H157, PC9 and H460 cells were treated with 0, 0.4, 4, 40, 400, or 4000 ng/ml of gemcitabine for 72 h. Cell viabilities were determined using CCK-8 assays **(*A*)**, and subG1 populations were quantified by PI staining and flow cytometry **(*B*)**. The values shown are mean percentages of cells relative to untreated cells in three independent experiments performed in triplicate; error bars represent SDs. ***C*, **A549, H157, PC9 and H460 cells were incubated in the presence of 0, 4, 40, 400, or 4000 ng/ml of gemcitabine for 24 h. Total RNAs were extracted and the mRNA levels of Bfl-1, Bcl-x_L_, Bcl-2, and β-actin were determined by RT-PCR. ***D*, **A549 and H157 cells were transiently transfected with pNF-κB-Luc (firefly) plasmid for 16 h, and subsequently treated with 0, 4, 40, 400, or 4000 ng/ml of gemcitabine for an additional 6 h. Cell were then lysed and analyzed for firefly luciferase activity. Data are mean luciferase activities normalized versus β-gal expression obtained from three independent experiments; error bars represent SDs. Results are presented as means ± SDs of three independent experiments performed in triplicate. *p < 0.001 by the Student's t test for difference in tumor volumes between experimental groups.

Initially, we examined the sensitivity of NSCLC cell lines (A549, H157, PC9 and H460) to gemcitabine using CCK-8 assays and subG1 analysis after incubating cells with 0, 0.4, 4, 40, 400, or 4000 ng/ml of gemcitabine for 72 h. High dose (4000 ng/ml) gemcitabine reduced cell viabilities by up to 50% and increased subG1 fractions to about 50%; however, low dose (4 to 40 ng/ml) gemcitabine decreased cell viability by only ≤ 10% and subG1 fractions to about 20% (Figures 1A and 1B), indicating that these lung cancer cells are relatively resistant to low dose gemcitabine.

Because Bcl-2 family members play a role in determining the sensitivity of cancer cells to gemcitabine [34,35], we examined the expressions of anti-apoptotic Bcl-2, Bcl-x_L_, and Bfl-1 by RT-PCR. As shown in Figure 1C, low dose gemcitabine markedly up-regulated Bfl-1 with little effect on Bcl-2 and Bcl-x_L_. At the concentration of 4000 ng/ml gemcitabine, where cell viability was markedly reduced, the Bfl-1 expression was decreased to the basal level in all cell lines except for H460 cells. Real-time RT-PCR analysis also demonstrated that low-dose gemcitabine led to up-regulation of Bfl-1 with no changes on the level of Bcl-2, Bcl-xL and Mcl-1 (Additional File 1, Figure S1).

NF-κB activation is associated with gemcitabine resistance, and Bfl-1 is a direct transcriptional target of NF-κB [36]. Therefore, to determine whether NF-κB is involved in the above-mentioned gemcitabine-induced up-regulation of Bfl-1, we evaluated NF-κB activities in A549 and H157 cells using luciferase assay. NF-κB activities were elevated by gemcitabine treatment at 4 to 400 ng/ml for 6 h (Figure 1D), which were relatively well correlated with the transcriptional induction of Bfl-1, as shown in Figure 1C. Of note, at a concentration of 4000 ng/ml which efficiently compromised cell viability (Figures 1A and 1B), NF-κB activity was returned to the basal level, which was consistent with the change of Bfl-1 expression (Figures 1C and 1D). These data suggest that lung cancer cells resist low dose gemcitabine via NF-κB activation and subsequent Bfl-1 overexpression.

The borderline dose of 400 ng/ml gemcitabine induces a decrease of cell viability whereas Bfl-1 and NF-kB are up-regulated. This discrepancy might result from the different time points of assays for each factor; namely, luciferase assay for NF-kB activity at 6 h after gemcitabine treatment, RNA extraction for Bfl-1 RT-PCR at 24 h, and CCK-8 assay for cell viability at 72 h. DNA damage caused by gemcitabine 400 ng/ml functions as a form of stress and can lead to the activation of NF-kB within 12 h [37]. However, in spite of initial activation of NF-kB, gemcitabine exerts delayed cytotoxic effects which can take longer than 72 h [38,39]

### The C-terminal region of Bfl-1 fused with GFP (BC) induced caspase-mediated apoptosis in lung cancer cells

**Figure 2:**
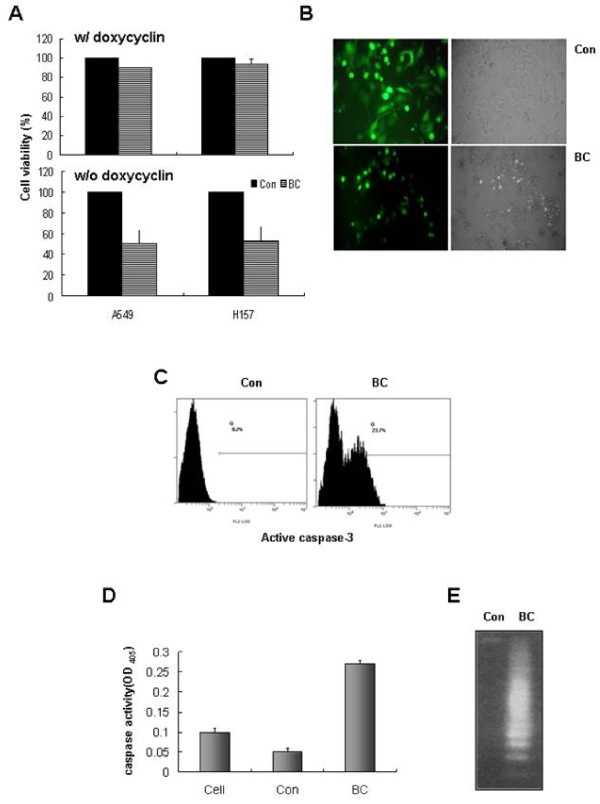
**C-terminal of Bfl-1 fused with GFP (BC) inhibits proliferation and induces lung cancer cell apoptosis**. ***A and B*, **A549 and H157 cells were infected with 10 MOI of the C-terminal of Bfl-1-GFP (BC) or control-GFP (Con) adenovirus and Tet-off adenovirus, and maintained with or without doxycycline for 72 h. In these environments, the expression of BC or GFP was inhibited in the presence of doxycycline and induced in its absence. Cell viabilities were determined using CCK-8 assays, and are expressed the mean percentages of control adenovirus infected cells as determined by three independent experiments. Error bars represent SDs ***(A)***. Cellular morphologies were examined by fluorescence microscopy ***(B)***. ***C, D and E*, **A549 cells were transfected with BC or Con and Tet-off adenoviruses, and cultured in the absence of doxycycline for 72 h. Active caspase-3 levels were measured by flow cytometry using PE-conjugated anti-active caspase-3 antibody ***(C)***. After infecting cells for 48 h, they were lysed and 100 μg of whole cell lysates were treated with caspase activity assay buffer containing the peptide caspase substrate DEVD-pNA, for 4 h. Results are the average absorbance at 405 nm of experiments conducted in triplicate, and error bars represent SDs ***(D)***. After infecting cells for 72 h, total genomic DNA was extracted and subjected to electrophoresis in 2% agarose gel to examine DNA fragmentation patterns ***(E)***.

We previously reported that a protein containing the C-terminal region of anti-apoptotic Bfl-1 and GFP is able to efficiently induce apoptosis in 293T cells [17,29]. In the present study, we utilized an adenoviral system for BC gene therapy in lung cancer cells and a Tet-inducible system, as described in Materials and Methods. Initially, we examined whether BC delivered via adenoviral vector could induce lung cancer cell apoptosis. Three days after infection, cell viabilities were assessed by fluorescence microscopy and CCK-8 assays. When A549 and H157 cells were co-infected with control (GFP) and Tet-off adenoviruses, more than 90% of the cells emitted green fluorescence, which suggested successful gene delivery by the adenovirus. Furthermore, whereas the control vector showed little cytotoxicity, the BC containing vector suppressed cell viability and induced cell death (Figures 2A and 2B). This cell death was thought to be apoptotic in nature, as indicated by the appearance of active caspase 3 and DNA laddering (Figures 2C, D and 2E). These findings indicate that BC is able to induce the apoptosis of lung cancer cells.

### BC restored lung cancer cell sensitivity to low dose gemcitabine

**Figure 3:**
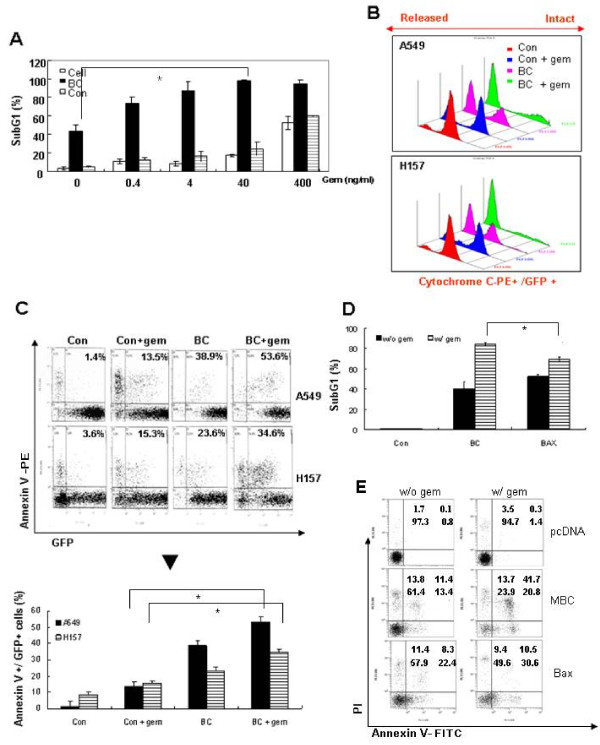
**BC sensitizes lung cancer cells to low dose gemcitabine**. ***A*, **A549 cells infected with BC (filled bars) or control (Con, hatched bars) adenovirus were treated with 0, 0.4, 4, 40, or 400 ng/ml of gemcitabine for 72 h. Cell deaths were determined by subG1 analysis. Open bars represent non-infected A549 cells. The graph was plotted using the means ± SDs of three experiments. *p < 0.001 by the Student's t test for difference in tumor volumes between experimental groups. ***B and C*, **A549 and H157 cells infected with BC or control adenovirus were cultured with or without 40 ng/ml gemcitabine for 72 h. Cells were analyzed by flow cytometry for cytochrome C release ***(B)***. Apoptotic cell death was determined by FACS analysis of annexin V-PE stained cells among GFP expressing cells. Representative data (upper) and means ± SDs of three experiments (lower) are shown. * *p *< 0.001 by the Student's t test for differences between amounts of annexin V-PE (+) cells by gemcitabine in control and BC infected cells **(C)**. ***D*, **A549 cells infected with BC (filled bars: BC only effect) or Bax (hatched bars) adenovirus were maintained in the presence or absence of 40 ng/ml gemcitabine for 72 h. SubG1 fractions were quantified by PI staining and FACS. Representative data (upper) and the means ± SDs of three experiments (lower) are shown. * *p *< 0.001 by the Student's t test for differences between amounts of subG1 fraction increases induced by gemcitabine in BC and Bax infected cells. ***E*, **A549 cells were transfected with pcDNA, pcDNA-MBC(no tagging GFP), or pcDNA-BAX; 24 hours later, the cells were stained with annexinV-FITC and PI, and cell death was analyzed by flow cytometry. Annexin V-FITC positive cell (without/with-gem), pCDNA: 0.8/1.4(%), MBC: 13.4/20.8(%) and BAX: 22.4/30.6(%). PI positive cell (without/with-gem): pCDNA: 1.7/3.5(%), MBC:13.8/13.7(%) and BAX:11.4/9.4(%). Annexin V-FITC/PI double positive cell (without/with-gem): pCDNA: 0.1/0.3(%), MBC: 11.4/41.7(%) and BAX: 8.3/10.5(%).

Next, we investigated whether BC could sensitize lung cancer cells to low dose gemcitabine. Commonly annexin V-FITC (green)/PI (red) is used to confirm apoptosis. However in this particular situation, the fluorescence emitted from GFP and FITC are both green and cannot be distinguished from each other. Therefore, instead of using annexin V-FITC, we used annexin-PE to obtain Figure 3C, and showed Annexin V/GFP + gating cells numbers in percentage by histogram. Whereas low dose (0.4 to 40 ng/ml) gemcitabine treatment for 72 h led to subG1 arrest in only 10-20% of A549 cells and BC monotherapy induced subG1 arrest in about 40% of cells, combined treatment with BC and low dose gemcitabine increased the subG1 population to 70-100% (Figure 3A). Cytochrome C release assays and annexin V staining also demonstrated that BC and low dose (40 ng/ml) gemcitabine acted additively or synergistically to cause mitochondria-mediated apoptosis in A549 and H157 cells (Figure 3B and 3C).

Because Bax gene therapy is widely used to induce chemosensitization and promote tumor cell apoptosis by disrupting mitochondrial membrane integrity [37], we compared the efficacies of BC and Bax gene therapies in combination with gemcitabine. As determined by subG1 analysis, BC was superior to Bax in sensitizing A549 cells to gemcitabine (*p < 0.001*) (Figure 3D and Figure Additional File 2, Figure S2). In addition, the C-terminal region of Bfl-1-fused with external charged residues from potential efficiency vector sequence (not GFP) (MBC) also showed proapoptotic function and sensitized A549 cells to gemcitabine (Figure 3E). Collectively, these findings suggest that A549 cells could be sensitized to low dose gemcitabine by co-administering BC via its additive or synergistic cytotoxic effect.

### BC suppressed NF-κB activity and down-regulated Bfl-1 expression, thereby sensitizing lung cancer cells to gemcitabine

**Figure 4:**
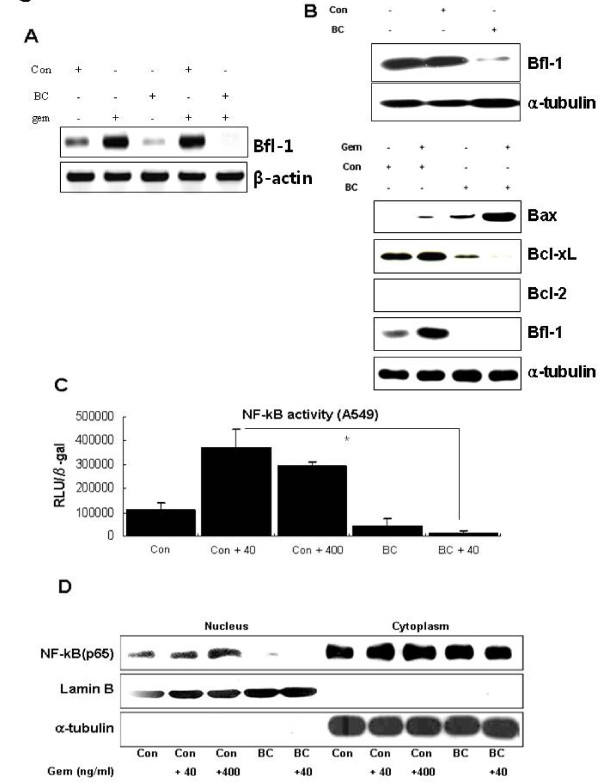
**BC down-regulates Bfl-1 expression by inhibiting NF-κB activity**. ***A*, **A549 cells infected by BC or control (Con) adenovirus were treated with 40 ng/ml gemcitabine for 72 h. Total RNA was isolated and subjected to RT-PCR for Bfl-1 and β-actin. ***B*, **A549 cells were infected with BC or control adenovirus and treated with 40 ng/ml gemcitabine or not. 50 μg of total cellular proteins were subjected to SDS-PAGE and then immunoblotted using anti-Bfl-1, -Bax, -Bcl-x_L_, -Bcl-2, and -α-tubulin antibodies. Data are representative of three independent experiments. ***C*, **A549 cells, transfected with the pNF-κB-Luc reporter gene and *β*-gal-lacZ plasmid, were infected with BC or control adenovirus and then treated with 40 or 400 ng/ml of gemcitabine for 48 h. Luciferase and β-galactosidase activities were determined using a luminometer. The values shown represent luciferase activities relative to β-gal, and the means ± SDs from three independent experiments are plotted. *p < 0.001 by the Student's t test for difference in tumor volumes between experimental groups. ***D*, **A549 cells infected with BC or control adenovirus were treated with 0, 40, or 400 ng/ml gemcitabine for 48 h. Proteins from nuclear and cytosolic fractions were separately isolated, and 50 μg aliquots were subjected to SDS-PAGE and then immunoblotted using anti-p65/relA, -lamin B, and -α-tubulin antibodies. Data are representative of three independent experiments.

Figure 1 and Figure Additional file 1, Figure S1 showed that low dose gemcitabine activated NF-κB and up-regulated Bfl-1 expression in lung cancer cells, which might be one of the underlying resistant mechanisms to low dose gemcitabine. Above observation that BC sensitized lung cancer cells to gemcitabine led us to investigate the effects of BC on NF-κB activity and Bfl-1 expression. As shown in Figure 4A, low dose (40 ng/ml) gemcitabine-induced Bfl-1 overexpression was inhibited by BC co-treatment in A549 cells. Bfl-1 protein expression was also suppressed by BC (Figure 4B). BC also down-regulated the Bcl- x_L _but increased the Bax expression (Figure 4B). These data suggested that BC might alter the balance of Bcl-2 family proteins towards the pro-apoptotic state, which includes down-regulation of Bfl-1. We next assessed the effect of BC on NF-κB activity in A549 cells. BC treatment was found to reduce NF-κB activity to half its basal level; furthermore, NF-κB activity, which had been elevated by more than 3-fold by gemcitabine at 40 ng/ml, markedly declined to a very low level when cells were co-treated with BC and gemcitabine (40 ng/ml) (Figure 4C). Immunoblotting also revealed that low dose gemcitabine caused the translocation of p65/relA from the cytoplasm to the nucleus, which was prevented by BC (Figure 4D). These findings together suggest that BC might suppress NF-κB activity and subsequently down-regulate Bfl-1, and thus, sensitizes lung cancer cells to gemcitabine-induced cell death.

**Figure 5:**
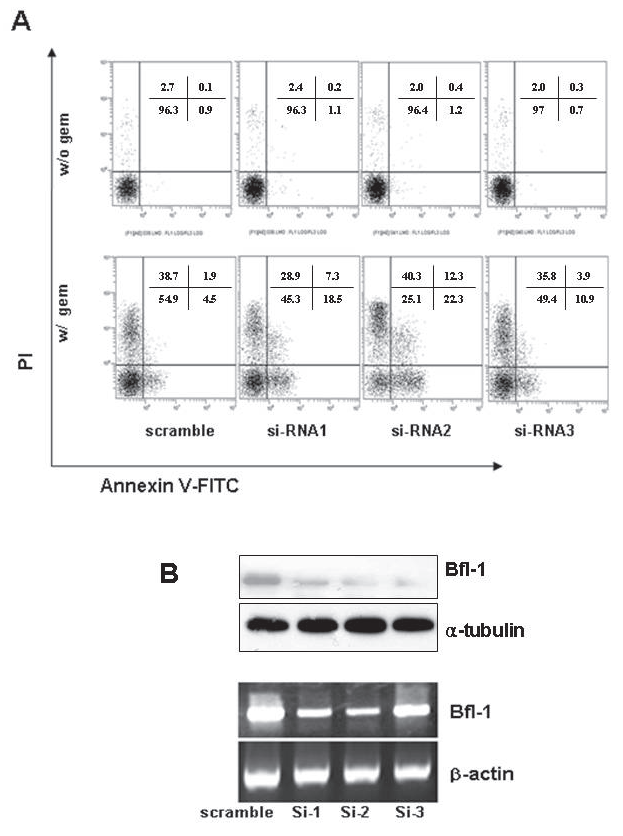
**Bfl-1 siRNA sensitized lung cancer cells to low dose gemcitabine**. ***A*, **A549 cells were transfected with three types of Bfl-1 siRNA and then treated with 40 ng/ml gemcitabine for 48 h. Cells were then stained with Annexin V-FITC/PI and analyzed by flow cytometry. Experiments were performed three times and yielded comparable results. ***B***, Total protein and RNA were extracted from A549 cells transfected with three types of Bfl-1 siRNA. 50 μg of total cellular proteins were subjected to SDS-PAGE and then immunoblotted using anti-Bfl-1 and -α-tubulin antibodies (upper panel), and RNAs were subjected to RT-PCR to determine Bfl-1 expression. β-actin was used as an internal control (lower panel).

However, it still remained unclear whether the BC-induced down-regulation of Bfl-1 caused the observed sensitization to gemcitabine or just was a coincidence. To address this, we examined whether the direct suppression of Bfl-1 by siRNA could sensitize A549 cells to gemcitabine-induced apoptosis. It was found that whereas the direct down-regulation of Bfl-1 had little effect on cell viability, Bfl-1 siRNA and gemcitabine co-treatment increased apoptotic cell death (Figures 5A and 5B). These data suggest that Bfl-1 might play a role in gemcitabine resistance of lung cancer cells, and that the down-regulation of Bfl-1, either directly or using BC, offers an effective means of making lung cancer cells sensitive to gemcitabine.

### BC and low dose gemcitabine co-treatment efficiently suppressed tumor growth and induced tumor cell death in a lung cancer xenograft model

**Figure 6:**
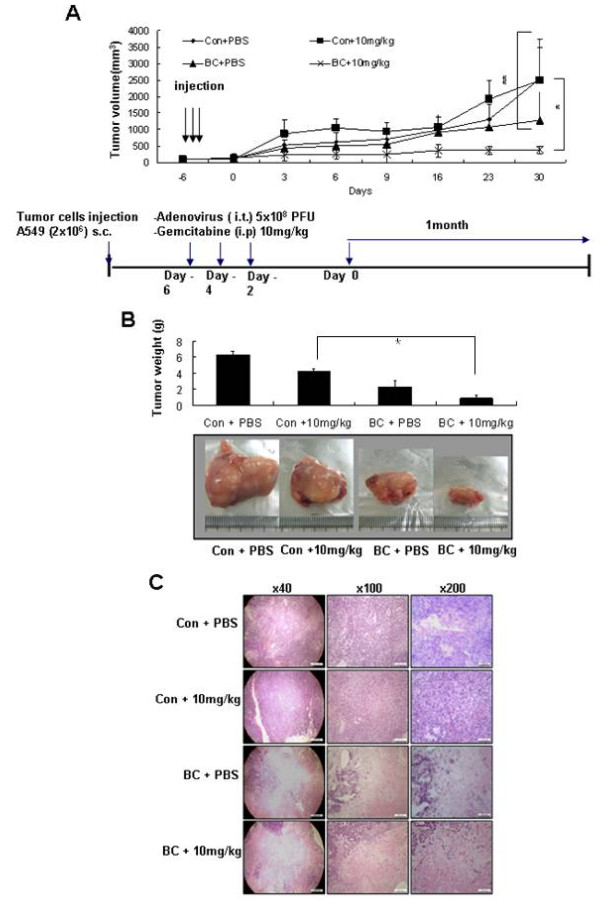
**Combined BC and low dose gemcitabine therapy efficiently suppressed tumor growth and induced tumor cell death in a lung cancer xenograft model**. ***A*, **A549 cells (2×10^6^) were injected into the flank of 6- to 8-week-old male nude mice to establish a tumor xenograft model. Three weeks later when tumor volumes had reached about 100 mm^3^, BC or control adenovirus and Tet-off adenovirus (5×10^6 ^PFU) were directly injected into tumors, and 10 mg/kg gemcitabine or PBS were administered intraperitoneally. These therapies were performed three times with two days intervals. Tumor volumes were measured every 2 to 3 days for one month. Each experimental group included 5 mice and means ± SDs are plotted. *p < 0.001 and ** *p *< 0.05 by the Student's t test for differences between tumor volumes between experimental groups. ***B and C*, **On day 30 after the last treatment, mice were sacrificed and tumors were extracted. The histogram shows the means ± SDs of tumor weights in each group (n = 5) ***(B)***. Tumor sections were analyzed by hematoxylin and eosin staining for histological features, such as, tumor necrosis and apoptosis. Numbers at the top of column indicates magnification ***(C)***.

Finally, to evaluate the *in vivo *anti-tumor effect of BC and low dose gemcitabine therapy, we established a human lung cancer xenograft model in nude mice using A549 cells as described in Materials and Methods. As shown in Figure 6, in mice treated with control adenovirus or gemcitabine, tumors reached mean volume of 2488 mm^3 ^± 1282 mm^3 ^at 30 days after the last treatment. However, tumor growth was significantly inhibited in mice co-treated with BC and gemcitabine; the average tumor volume was 380 ± 97 mm^3 ^at 30 days (a reduction of 85% versus control mice treated with adenovirus and PBS), which consistent with more than an 80% reduction in tumor weight (Figures 6A and 6B). Histologic examination of tumors revealed more extensive necrosis and frequent apoptotic cells with nuclear condensation and fragmentation in mice co-treated with BC and gemcitabine than in control mice or those treated with gemcitabine only (Figure 6C and Additional File 3, Figure S3). These findings suggest that co-treatment with BC and low dose gemcitabine could efficiently suppress tumor growth and induced tumor cell death *in vivo*.

## Discussion

Gemcitabine has been previously shown to be effective in NSCLC, but acquired resistance after prolonged and repeated exposure was found to reduce response rates in clinical trials [7,40]. Moreover, because high therapeutic doses of gemcitabine cause bone marrow suppression and reduce general health status, optimal dosages and medication schedules are required to achieve tumor control and improve quality of life [41]. For example, low doses of gemcitabine could be continuously administered by infusion-based to patients with locally advanced disseminated cancer, because of its lower toxicity than the standard gemcitabine regimen [42]. However, chemotherapeutic stresses elicited by low concentrations of gemcitabine lead to NF-κB activation and enhance Akt signaling, and thereby cause drug resistance [43]. Otherwise, in practice, to overcome low response rates in NSCLC, gemcitabine is used in combination with other cytostatic agents with different modes of action [44]. Therefore, it is conceivable that combinational approaches based on gene therapy targeting the drug resistance pathway and gemcitabine, as shown by the present study, might have a merit for tumor control.

NF-κB activation status of cancer cells can be represented by its basal level or chemotherapy-induced up-regulation of NF-κB activity. Which of them is more important for chemoresistance in cancer remains controversial [45]. In previous reports, NF-κB activity was high at the basal level, and was also elevated by gemcitabine treatment in pancreatic cancer, breast cancer, and NSCLC. NF-κB activation and the subsequent up-regulations of NF-κB-regulated genes have been suggested to attenuate the efficiency of gemcitabine. In fact, several studies have reported that the suppression of NF-κB activity can sensitize cancer cells to gemcitabine [5,6,18]. On the other hand, Pan et al. recently reported that there was no significant correlation between basal NF-κB activity and gemcitabine sensitivity, and that gemcitabine did not activate NF-κB in pancreatic cancer cells [5]. Furthermore, silencing of p65/relA increased apoptosis in gemcitabine-sensitive pancreatic cancer cells, but not in resistant cells [19]. These conflicting observations suggest that the association between gemcitabine efficacy and NF-κB activity might not be identical among cancers, but should be considered in a cell type-specific manner.

In agreement with previous studies by Denlinger et al. using NSCLC cell lines (A549 and H157) [18], we observed that low dose gemcitabine enhanced NF-κB activity in A549 and H157 cells. In line with the gemcitabine-induced NF-κB activation, its target gene Bfl-1 was found to be markedly up-regulated, with little effect on the levels of other anti-apoptotic Bcl-2 proteins, including Bcl-2 and Bcl-x_L_; furthermore, this was observed in all NSCLC cell lines examined. Bfl-1overexpression has been reported to underlie resistance to various apoptotic stimuli [23], and we previously observed that Bfl-1 is frequently over-expressed in lung cancer cell lines [28]. Therefore, we speculated that Bfl-1 might regulate the sensitivity of NSCLC to gemcitabine. As was expected, direct Bfl-1 suppression using siRNA sensitized cells to gemcitabine-induced apoptosis in A549 cells. In a study conducted by Brien et al. in B lymphoblastoid and diffuse large B-cell lymphoma cell lines, Bfl-1 silencing was found to potently induce apoptosis, and sensitize cells to rituximab-mediated cell death and apoptosis by doxorubicin, vincristine, cisplatin, or fludarabine [25]. In the present study using lung cancer cell lines, although siRNA-mediated Bfl-1 suppression *per se *did not affect cell viability, it sensitized cells to gemcitabine. Therefore, the present and our previous findings suggest that Bfl-1 is a feasible molecular target for enhancing the efficacy of gemcitabine in lung cancer. Furthermore, in view of the fact that BC itself alone has a cytotoxic effect, it would appear that BC is likely to be better than siRNA at chemosensitizing cells. Although previous studies have reported that NF-κB regulates the expression of Bcl-xL and/or Bcl-2 in various cell types [46], we observe little changes in the level of Bcl-xL and Bcl-2 in cells treated with gemcitabine at low concentrations inducing NF-κB activation either by RT-PCR and real-time RT-PCR. It is possible that Bfl-1, a direct target of NF--κB, might function as an important and sensitive anti-apoptotic Bcl-2 family protein reflecting the alteration of NF--κB activity in NSCLC cells, particularly in terms of response to gemcitabine.

Although Bfl-1 is an anti-apoptotic bcl-2 family member and primarily prevents apoptosis, its protective effect depends on cell type and apoptotic stimulus [26,27,47]. Furthermore, Bfl-1 can be converted into a pro-apoptotic molecule when processed by proteasome or TNF receptor signaling in pro-B cells [27,47]. In this system, the Bfl-1 C-terminal domain was important for the pro-apoptotic function of Bfl-1, because it was required for Bfl-1 ubiquitination and its localization at the mitochondrial membrane[29]. Our group, previously, demonstrated that anti-apoptotic Bfl-1 is converted to a pro-apoptotic protein when fused with GFP, and that the 29 amino acids of its C-terminal region are sufficient to induce apoptotic cell death via the mitochondrial pathway and caspase activation in 293T cells [17,29,48]. An amphipathic tail-anchoring domain of the Bfl-1 C-terminal region has been implicated in the targeting of Bfl-1 to the mitochondrial membrane to induce apoptosis [17,27,47]. These findings led us to hypothesize that the Bfl-1 C-terminal region fused with GFP (BC) could be harnessed as a gene therapy in combination with chemotherapeutic agents to achieve management of cancer.

Previously, we used a plasmid vector system to deliver BC to 293T cells to investigate its apoptotic effects. However, BC-plasmid showed a little cytotoxicity in A549 cells as compared in 293T cells, which was considered to be due to low transfection efficiencies in the cancer cell lines examined. Therefore, in the present study, we utilized an adenoviral system to examine the possibilities of BC gene therapy. Accordingly, we generated a replication-deficient adenovirus expressing BC, GFP only (Control), or Bax-GFP, and employed a Tet-inducible system. As was expected, transfection of BC using this adenoviral system efficiently induced apoptosis in A549 and H157 cells. Moreover, we found that BC remarkably suppressed NF-κB activity. Although transfection with pro-apoptotic Bax induced cell death at higher levels than BC alone, BC sensitized cells to gemcitabine more than Bax, which we attribute to the NF-κB suppressing effect of BC. Taken together, we consider that BC inhibits NF-κB and subsequently down-regulates Bfl-1, thereby sensitizing cells to gemcitabine-induced apoptosis in an additive or synergistic manner. We also found that co-treatment with BC delivered by adenovirus using a Tet-inducible system in combination with low dose gemcitabine efficiently inhibited tumor growth and induced tumor cell apoptosis and necrosis with little systemic toxicity in a xenograft mouse model. These results cautiously raise the possibility that BC gene therapy could be used to lower gemcitabine doses in order to control cancer and avoid toxic side effects. Because its intrinsic cytotoxic effect of BC, we were cautious to reach a conclusion that BC sensitized lung cancer cells to gemcitabine-induced cell death in a synergistic rather than just additive manner. Although the results of apoptosis assay by FACS (Figure 3C) showed an additive cytotoxic effect of BC and gemcitabine rather than synergistic, subG1 analysis (Figure 3D) and in vivo tumor suppressive effect (Figure 6A) of BC and gemcitabine was synergistic rather than additive.

The way how BC suppresses NF-κB activity remains to be elucidated, and if BC could directly down-regulated Bfl-1 by mechanisms other than inhibition of NF-κB is open to further study. We currently just have some speculations on this subject. One of them is that because Bfl-1 is constitutively ubiquitinized at its C-terminal region and processed by proteasomal degradation, BC might function as a kind of competitive inhibitor of proteasome, thereby leading to NF-κB suppression and cell death. We have a clue that p53 pathway might be involving; we have observed that BC causes NF-κB inhibition through p53 activation (data not shown). However, more precise mechanism should be clarified by further study.

BC and low dose gemcitabine also showed synergistic cytotoxic effects in the MDA-MB-231 and MCF7 breast cancer cell lines (Additional file 4, Figures S4A and B). Furthermore, to test if BC can overcome resistance to high dose gemcitabine, we established acquired gemcitabine resistant cell lines, A549GR, which resist gemcitabine up to 40000 ng/ml, and included intrinsic gemcitabine-resistant pancreas cancer cell lines, Panc1. As shown in Additional file 4, Figure S4A, BC co-treatment induced cell death in high dose gemcitabine-resistant cells. We also observed that BC was able to sensitize A549 and H157 cells to sublethal doses of etoposide, doxorubicin, or staurosporine again in an additive or synergistic manner (Additional File 4, Figure S4C). These data suggests that BC gene therapy might be used to overcome resistance to chemotherapeutic agents other than gemcitabine in variable cancers. However, our main focuses were gemcitabine- NF-κB -Bfl-1 axis implicated in low gemcitabine resistance of lung cancer cells, and the utility of BC to overcome gemcitabine resistance in this system. Therefore we tried to show the efficient of combined BC and gemcitabine therapy and to dissect the underlying mechanism. If the same axis of gemcitabine- NF-κB -Bfl-1 regulation would be working in the above mentioned additive or synergistic cytotoxicity of BC in other system remains to be clarified.

Summarizing, the present study demonstrates that lung cancer cells resist low dose gemcitabine because NF-κB is activated by gemcitabine and Bfl-1 is subsequently up-regulated. BC transfection was found to sensitize cells to gemcitabine by suppressing NF-κB activity and Bfl-1; and moreover co-treatment by BC transfection and gemcitabine chemotherapy showed a strong anti-tumor effect *in vivo*. Our findings indicate that targeting the NF--κB/Bfl-1 pathway with BC might be utilized for improving the chemotherapeutic effects of gemcitabine in lung cancer.

## Abbreviations List

BC: C-terminal region of Bfl-1 fused with GFP; NSCLC: non-small cell lung cancer; siRNA: small interfering RNA.

## Competing interests

The authors declare that they have no competing interests.

## Authors' contributions

MKK performed most of the experiments and worked together with YKJ to analyze the data and prepare and write the manuscript. JKW and YC participated in vivo-experiment. DHC carried out the polyclonal Bfl-1 antibody manufacture. YHK participated in coordination of the study. CWK contributed to the design of the project, to data analysis, and to writing of the paper. All authors reviewed and approved the final manuscript.

## Financial Support

This work was supported by KOSEF through the Tumor Immunity Medical Research Center (TIMRC) at Seoul National University College of Medicine.

## Supplementary Material

Additional file 1 **Figure S1. Low dose gemcitabine markedly up-regulated Bfl-1 mRNA level**. Total RNA was isolated from cells using TRIzol reagent (Invitrogen) according to the manufacturer's instructions. 5 μg aliquots of total RNA were reversely transcribed to cDNAs using a RT-PCR kit (Promega, Madison, WI). Then real-time PCR was carried out using Power SYBR^® ^Green PCR Master Mix (Applied Biosystems, Foster, CA) with a Bio-Rad iCycler iQ system, and gene-specific primers as follows; Bfl-1, forward 5'-CAGCACATTGAATCAACAGC-3' and reverse 5'-TGCAGATAGTCCTGAGCCAGC-3'; Bcl-xL, forward 5'-GAGGCAGGCGACGAGTTTGAA-3' and reverse 5'-GGGGTGGGAGGGTAGAGTGGA-3'; Mcl-1, forward 5'-AAGCCAATGGGCAGGTCT-3' and reverse 5'-TGTCCAGTTTCCGAAGCAT-3'; β-actin, forward 5'-GGAAATCGTGCGTGACATTAAGG-3' and reverse 5'-GGCTTTTAGGATGGCAAGGGAC-3'. Each sample was run in triplicate. Relative transcript abundance was calculated using the comparative C_T _method for a standard control β-actin. Thermal cycling conditions were as follows: 95°C for 3 min, followed by 40 cycles of 95°C for 15 s and 55°C for 30 s. Click here for file

Additional file 2 **Figure S2. BC was better in sensitizing lung cancer cells to gemcitabine than Bax**. A549 cells infected with BC or BAX adenovirus were maintained in the presence or absence of 40 ng/ml gemcitabine for 72 h. SubG1 fractions were quantified by PI staining and FACS. GFP (%) represented infection efficiency of adenovirus constructs. Click here for file

Additional file 3 **Figure S3. Staining for Histological features**. On day 30 after the last treatment, mice were sacrificed and tumors were extracted. The histogram shows the means ± SDs of tumor weights in each group (n = 5). Tumor sections were analyzed by hematoxylin and eosin staining for histologic features, such as, tumor necrosis and apoptosis. Numbers at the top of column indicates magnification. Click here for file

Additional file 4 **Figure S4. BC synergistically compromised cell viability when administered with different chemotherapeutics to cancer cell lines**. Human breast cancer cell lines, MDA-MB-231 (ATCC HTB-26, passage-8) and MCF7 (ATCC HTB-22, passage-13) and PANC-1(ATCC CRL-1469, passage-5) cells were verified by morphology, growth curve analysis and tested for *Mycoplasma*. All cell lines were obtained from and characterized by the American Type Culture Collection (ATCC). No further authentication was done by the authors. Etoposide, doxorubicin and staurosporine were purchased from LC Laboratories (Woburn, MA). ***A*, **MDA-MB-231 (MDA) and MCF7 (MCF) (human breast cancer cell lines), A549, H460, PC9 (human lung adenocarcinoma cell lines), and H157 (a human lung squamous cell carcinoma cell line) cells infected by BC or control adenovirus and Tet-off adenovirus, and then treated with 40 ng/ml gemcitabine for 72 h ***(upper panel)***. A549, gemcitabine-resistant A549 cells (A549GR), and PANC-1 (an intrinsic gemcitabine-resistant human pancreatic carcinoma cell line) were cells were infected with BC or control adenovirus and Tet-off adenovirus, and then treated with 0, 40, or 40000 ng/ml gemcitabine for 72 h ***(lower panel)***. Gemcitabine-resistant A549 cells (A549GR) were generated by exposure to increasing concentrations of gemcitabine from the first concentration of 40 ng/ml to final selection concentration of 4μg/ml. A549GR cells were passaged 4 to 5 times in the absence of drug about for 2 weeks, and maintained in the final selection concentration. Cell viabilities were measured using CCK-8 assays. Values are expressed as mean percentages of untreated cells in three independent experiments performed in triplicate; error bars represent SDs. ***B*, **A549, H157, H460 and MDA-MB-231 cells infected by BC or control adenovirus and Tet-off adenovirus treated with 40 ng/ml gemcitabine for 72 h. Cytochrome C release was analyzed by flow cytometry. Values are expressed as mean percentages of untreated cells in three independent experiments performed in triplicate; error bars represent SDs. ***C*, **A549 and H157 cells infected by BC or control adenovirus and Tet-off adenovirus were treated with 40 ng/ml gemcitabine, 10 nM etoposide (eto), 10 nM doxorubicin (dox), or 1 nM staurosporine (sts) for 72 h. Cell viabilities were determined using CCK-8 assays and are expressed relative to numbers of untreated cells. Results are presented as means ± SDs of three independent experiments performed in triplicate. *p < 0.001 by the Student's t test for differences between tumor volumes between experimental groups. Click here for file

## References

[B1] Cascone T Gridelli C Ciardiello F Combined targeted therapies in non-small cell lung cancer: a winner strategy? Curr Opin Oncol 2007 19 98 102 10.1097/CCO.0b013e328011beec 17272980

[B2] Marx G Harper P Non-platinum gemcitabine combinations in non-small cell lung cancer Lung Cancer 2002 38 Suppl 2 S51 54 12431830 10.1016/s0169-5002(02)00358-6

[B3] Crino L Cappuzzo F Gemcitabine in non-small cell lung cancer Expert Opin Pharmacother 2002 3 745 753 10.1517/14656566.3.6.745 12036414

[B4] Scagliotti GV Novello S Gemcitabine (Gemzar)-based induction chemotherapy in non-small-cell lung cancer Lung Cancer 2002 38 Suppl 2 S13 19 12431824 10.1016/s0169-5002(02)00352-5

[B5] Arlt A Gehrz A Muerkoster S Vorndamm J Kruse ML Folsch UR Schafer H Role of NF-kappaB and Akt/PI3K in the resistance of pancreatic carcinoma cell lines against gemcitabine-induced cell death Oncogene 2003 22 3243 3251 10.1038/sj.onc.1206390 12761494

[B6] Hernandez-Vargas H Rodriguez-Pinilla SM Julian-Tendero M Sanchez-Rovira P Cuevas C Anton A Rios MJ Palacios J Moreno-Bueno G Gene expression profiling of breast cancer cells in response to gemcitabine: NF-kappaB pathway activation as a potential mechanism of resistance Breast Cancer Res Treat 2007 102 157 172 10.1007/s10549-006-9322-9 17039268

[B7] Bergman AM Pinedo HM Peters GJ Determinants of resistance to 2',2'-difluorodeoxycytidine (gemcitabine) Drug Resist Updat 2002 5 19 33 10.1016/S1368-7646(02)00002-X 12127861

[B8] Okamoto K Ocker M Neureiter D Dietze O Zopf S Hahn EG Herold C bcl-2-specific siRNAs restore gemcitabine sensitivity in human pancreatic cancer cells J Cell Mol Med 2007 11 349 361 10.1111/j.1582-4934.2007.00013.x 17378914 PMC3822833

[B9] Achiwa H Oguri T Sato S Maeda H Niimi T Ueda R Determinants of sensitivity and resistance to gemcitabine: the roles of human equilibrative nucleoside transporter 1 and deoxycytidine kinase in non-small cell lung cancer Cancer Sci 2004 95 753 757 10.1111/j.1349-7006.2004.tb03257.x 15471562 PMC11158492

[B10] Davidson JD Ma L Flagella M Geeganage S Gelbert LM Slapak CA An increase in the expression of ribonucleotide reductase large subunit 1 is associated with gemcitabine resistance in non-small cell lung cancer cell lines Cancer Res 2004 64 3761 3766 10.1158/0008-5472.CAN-03-3363 15172981

[B11] Bergman AM Eijk PP Ruiz van Haperen VW Smid K Veerman G Hubeek I van den Ijssel P Ylstra B Peters GJ In vivo induction of resistance to gemcitabine results in increased expression of ribonucleotide reductase subunit M1 as the major determinant Cancer Res 2005 65 9510 9516 10.1158/0008-5472.CAN-05-0989 16230416

[B12] Dumontet C Bauchu EC Fabianowska K Lepoivre M Wyczechowska D Bodin F Rolland MO Common resistance mechanisms to nucleoside analogues in variants of the human erythroleukemic line K562 Adv Exp Med Biol 1999 457 571 577 10500836 10.1007/978-1-4615-4811-9_63

[B13] Galmarini CM Clarke ML Jordheim L Santos CL Cros E Mackey JR Dumontet C Resistance to gemcitabine in a human follicular lymphoma cell line is due to partial deletion of the deoxycytidine kinase gene BMC Pharmacol 2004 4 8 10.1186/1471-2210-4-8 15157282 PMC428575

[B14] Guchelaar HJ Vermes A Vermes I Haanen C Apoptosis: molecular mechanisms and implications for cancer chemotherapy Pharm World Sci 1997 19 119 125 10.1023/A:1008654316572 9259027

[B15] Wang CY Cusack JC Jr Liu R Baldwin AS Jr Control of inducible chemoresistance: enhanced anti-tumor therapy through increased apoptosis by inhibition of NF-kappaB Nat Med 1999 5 412 417 10.1038/7410 10202930

[B16] Jones DR Broad RM Comeau LD Parsons SJ Mayo MW Inhibition of nuclear factor kappaB chemosensitizes non-small cell lung cancer through cytochrome c release and caspase activation J Thorac Cardiovasc Surg 2002 123 310 317 10.1067/mtc.2002.118684 11828291

[B17] Ko JK Choi KH Kim HJ Choi HY Yeo DJ Park SO Yang WS Kim YN Kim CW Conversion of Bfl-1, an anti-apoptotic Bcl-2 family protein, to a potent pro-apoptotic protein by fusion with green fluorescent protein (GFP) FEBS Lett 2003 551 29 36 10.1016/S0014-5793(03)00872-X 12965200

[B18] Denlinger CE Rundall BK Keller MD Jones DR Proteasome inhibition sensitizes non-small-cell lung cancer to gemcitabine-induced apoptosis Ann Thorac Surg 2004 78 1207 1214 discussion 1207-1214 10.1016/j.athoracsur.2004.04.029 15464472

[B19] Pan X Arumugam T Yamamoto T Levin PA Ramachandran V Ji B Lopez-Berestein G Vivas-Mejia PE Sood AK McConkey DJ Logsdon CD Nuclear factor-kappaB p65/relA silencing induces apoptosis and increases gemcitabine effectiveness in a subset of pancreatic cancer cells Clin Cancer Res 2008 14 8143 8151 10.1158/1078-0432.CCR-08-1539 19088029 PMC4403242

[B20] Schniewind B Christgen M Kurdow R Haye S Kremer B Kalthoff H Ungefroren H Resistance of pancreatic cancer to gemcitabine treatment is dependent on mitochondria-mediated apoptosis Int J Cancer 2004 109 182 188 10.1002/ijc.11679 14750167

[B21] Bold RJ Chandra J McConkey DJ Gemcitabine-induced programmed cell death (apoptosis) of human pancreatic carcinoma is determined by Bcl-2 content Ann Surg Oncol 1999 6 279 285 10.1007/s10434-999-0279-x 10340887

[B22] Hopkins-Donaldson S Cathomas R Simoes-Wust AP Kurtz S Belyanskaya L Stahel RA Zangemeister-Wittke U Induction of apoptosis and chemosensitization of mesothelioma cells by Bcl-2 and Bcl-xL antisense treatment Int J Cancer 2003 106 160 166 10.1002/ijc.11209 12800189

[B23] Kim JK Kim KD Lee E Lim JS Cho HJ Yoon HK Cho MY Baek KE Park YP Paik SG Up-regulation of Bfl-1/A1 via NF-kappaB activation in cisplatin-resistant human bladder cancer cell line Cancer Lett 2004 212 61 70 10.1016/j.canlet.2004.02.021 15246562

[B24] Zong WX Edelstein LC Chen C Bash J Gelinas C The prosurvival Bcl-2 homolog Bfl-1/A1 is a direct transcriptional target of NF-kappaB that blocks TNFalpha-induced apoptosis Genes Dev 1999 13 382 387 10.1101/gad.13.4.382 10049353 PMC316475

[B25] Brien G Trescol-Biemont MC Bonnefoy-Berard N Downregulation of Bfl-1 protein expression sensitizes malignant B cells to apoptosis Oncogene 2007 26 5828 5832 10.1038/sj.onc.1210363 17353899

[B26] Wang CY Guttridge DC Mayo MW Baldwin AS Jr NF-kappaB induces expression of the Bcl-2 homologue A1/Bfl-1 to preferentially suppress chemotherapy-induced apoptosis Mol Cell Biol 1999 19 5923 5929 10454539 10.1128/mcb.19.9.5923 PMC84448

[B27] Kucharczak JF Simmons MJ Duckett CS Gelinas C Constitutive proteasome-mediated turnover of Bfl-1/A1 and its processing in response to TNF receptor activation in FL5.12 pro-B cells convert it into a prodeath factor Cell Death Differ 2005 12 1225 1239 10.1038/sj.cdd.4401684 16094403

[B28] Ko JK Lee MJ Cho SH Cho JA Lee BY Koh JS Lee SS Shim YH Kim CW Bfl-1S, a novel alternative splice variant of Bfl-1, localizes in the nucleus via its C-terminus and prevents cell death Oncogene 2003 22 2457 2465 10.1038/sj.onc.1206274 12717423

[B29] Yang WS Ko JK Park SO Choi HY Kim YN Kim CW C-terminal region of Bfl-1 induces cell death that accompanies caspase activation when fused with GFP J Cell Biochem 2005 94 1234 1247 10.1002/jcb.20381 15696550

[B30] Kim E Kim JH Shin HY Lee H Yang JM Kim J Sohn JH Kim H Yun CO Ad-mTERT-delta19, a conditional replication-competent adenovirus driven by the human telomerase promoter, selectively replicates in and elicits cytopathic effect in a cancer cell-specific manner Hum Gene Ther 2003 14 1415 1428 10.1089/104303403769211637 14577922

[B31] Triezenberg SJ LaMarco KL McKnight SL Evidence of DNA: protein interactions that mediate HSV-1 immediate early gene activation by VP16 Genes Dev 1988 2 730 742 10.1101/gad.2.6.730 2843426

[B32] Triezenberg SJ Kingsbury RC McKnight SL Functional dissection of VP16, the trans-activator of herpes simplex virus immediate early gene expression Genes Dev 1988 2 718 729 10.1101/gad.2.6.718 2843425

[B33] Waterhouse NJ Trapani JA A new quantitative assay for cytochrome c release in apoptotic cells Cell Death Differ 2003 10 853 855 10.1038/sj.cdd.4401263 12815469

[B34] Ng SS Tsao MS Nicklee T Hedley DW Wortmannin inhibits pkb/akt phosphorylation and promotes gemcitabine antitumor activity in orthotopic human pancreatic cancer xenografts in immunodeficient mice Clin Cancer Res 2001 7 3269 3275 11595724

[B35] Rieger J Durka S Streffer J Dichgans J Weller M Gemcitabine cytotoxicity of human malignant glioma cells: modulation by antioxidants, BCL-2 and dexamethasone Eur J Pharmacol 1999 365 301 308 10.1016/S0014-2999(98)00883-8 9988115

[B36] Cheng Q Lee HH Li Y Parks TP Cheng G Upregulation of Bcl-x and Bfl-1 as a potential mechanism of chemoresistance, which can be overcome by NF-kappaB inhibition Oncogene 2000 19 4936 4940 10.1038/sj.onc.1203861 11039911

[B37] Boland MP Fitzgerald KA O'Neill LA Topoisomerase II is required for mitoxantrone to signal nuclear factor kappa B activation in HL60 cells The Journal of biological chemistry 2000 275 25231 25238 10.1074/jbc.275.33.25231 10940316

[B38] Santini V Bernabei A Gozzini A Scappini B Zoccolante A D'Ippolito G Figuccia M Ferrini PR Apoptotic and antiproliferative effects of gemcitabine and gemcitabine plus Ara-C on blast cells from patients with blast crisis chronic myeloproliferative disorders Haematologica 1997 82 11 15 9172997

[B39] Pace E Melis M Siena L Bucchieri F Vignola AM Profita M Gjomarkaj M Bonsignore G Effects of gemcitabine on cell proliferation and apoptosis in non-small-cell lung cancer (NSCLC) cell lines Cancer chemotherapy and pharmacology 2000 46 467 476 10.1007/s002800000183 11138460

[B40] Carmichael J The role of gemcitabine in the treatment of other tumours Br J Cancer 1998 78 Suppl 3 21 25 9717987 10.1038/bjc.1998.750 PMC2062798

[B41] Takeuchi N Maejima S Hasebe O Matsuda Y Hanazaki K Kajikawa S Mukawa K Hosokawa K Hayashi K Hisa T [Clinical problems in gemcitabine treatment for unresectable pancreatic cancer in the elderly--a multicentric retrospective study of 53 cases] Gan To Kagaku Ryoho 2004 31 1987 1991 15570926

[B42] Sakamoto H Kitano M Suetomi Y Takeyama Y Ohyanagi H Nakai T Yasuda C Kudo M Comparison of standard-dose and low-dose gemcitabine regimens in pancreatic adenocarcinoma patients: a prospective randomized trial J Gastroenterol 2006 41 70 76 10.1007/s00535-005-1724-7 16501860

[B43] Meng F Henson R Patel T Chemotherapeutic stress selectively activates NF-kappa B-dependent AKT and VEGF expression in liver cancer-derived endothelial cells Am J Physiol Cell Physiol 2007 293 C749 760 10.1152/ajpcell.00537.2006 17537803

[B44] Abratt RP Sandler A Crino L Steward WP Shepherd FA Green MR Nguyen B Peters GJ Combined cisplatin and gemcitabine for non-small cell lung cancer: influence of scheduling on toxicity and drug delivery Semin Oncol 1998 25 35 43 9728583

[B45] Arlt A Schafer H NFkappaB-dependent chemoresistance in solid tumors Int J Clin Pharmacol Ther 2002 40 336 347 12467302 10.5414/cpp40336

[B46] Ko JK Choi KH Peng J He F Zhang Z Weisleder N Lin J Ma J Amphipathic tail-anchoring peptide and Bcl-2 homology domain-3 (BH3) peptides from Bcl-2 family proteins induce apoptosis through different mechanisms J Biol Chem 2011 286 9038 9048 10.1074/jbc.M110.198457 21189256 PMC3059050

[B47] Werner AB de Vries E Tait SW Bontjer I Borst J Bcl-2 family member Bfl-1/A1 sequesters truncated bid to inhibit is collaboration with pro-apoptotic Bak or Bax J Biol Chem 2002 277 22781 22788 10.1074/jbc.M201469200 11929871

[B48] Ko JK Choi KH Pan Z Lin P Weisleder N Kim CW Ma J The tail-anchoring domain of Bfl1 and HCCS1 targets mitochondrial membrane permeability to induce apoptosis J Cell Sci 2007 120 2912 2923 10.1242/jcs.006197 17666431

