# Supplementary figures and images for: The C-terminal region of Bfl-1 sensitizes non-small cell lung cancer to gemcitabine-induced apoptosis by suppressing NF-κB activity and down-regulating Bfl-1
Source: Mol Cancer. 2011 Aug 16;10:98. doi: 10.1186/1476-4598-10-98 (PMC3166274; doi:10.1186/1476-4598-10-98)

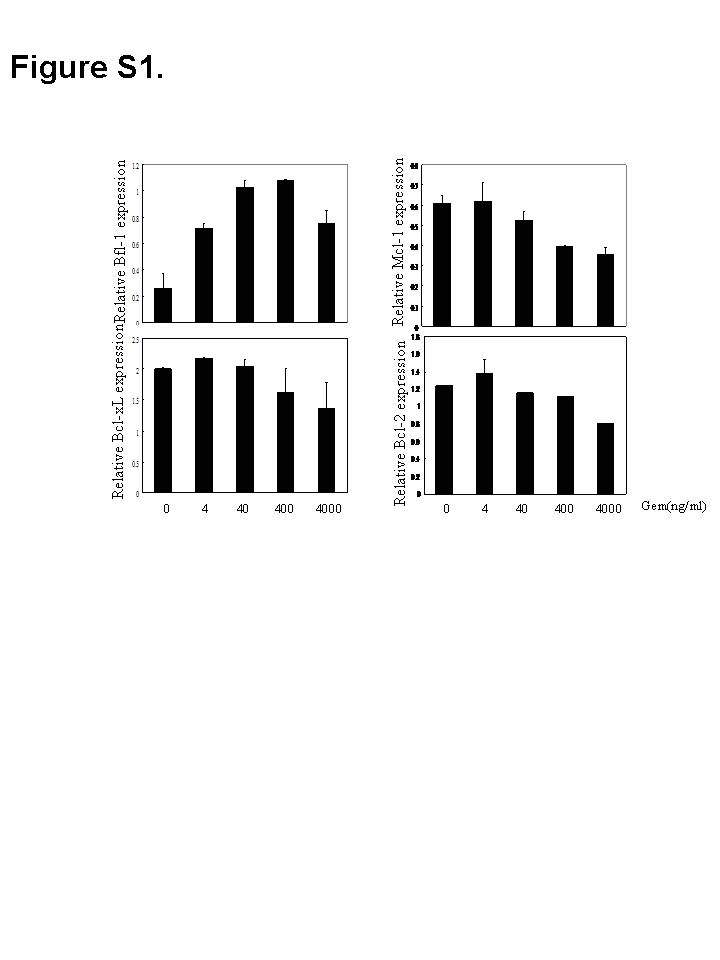

Supplement: Additional file 1 — Figure S1. Low dose gemcitabine markedly up-regulated Bfl-1 mRNA level. Total RNA was isolated from cells using TRIzol reagent (Invitrogen) according to the manufacturer's instructions. 5 μg aliquots of total RNA were reversely transcribed to cDNAs using a RT-PCR kit (Promega, Madison, WI). Then real-time PCR was carried out using Power SYBR® Green PCR Master Mix (Applied Biosystems, Foster, CA) with a Bio-Rad iCycler iQ system, and gene-specific primers as follows; Bfl-1, forward 5'-CAGCACATTGAATCAACAGC-3' and reverse 5'-TGCAGATAGTCCTGAGCCAGC-3'; Bcl-xL, forward 5'-GAGGCAGGCGACGAGTTTGAA-3' and reverse 5'-GGGGTGGGAGGGTAGAGTGGA-3'; Mcl-1, forward 5'-AAGCCAATGGGCAGGTCT-3' and reverse 5'-TGTCCAGTTTCCGAAGCAT-3'; β-actin, forward 5'-GGAAATCGTGCGTGACATTAAGG-3' and reverse 5'-GGCTTTTAGGATGGCAAGGGAC-3'. Each sample was run in triplicate. Relative transcript abundance was calculated using the comparative CT method for a standard control β-actin. Thermal cycling conditions were as follows: 95°C for 3 min, followed by 40 cycles of 95°C for 15 s and 55°C for 30 s. [file 1476-4598-10-98-S1.JPEG]

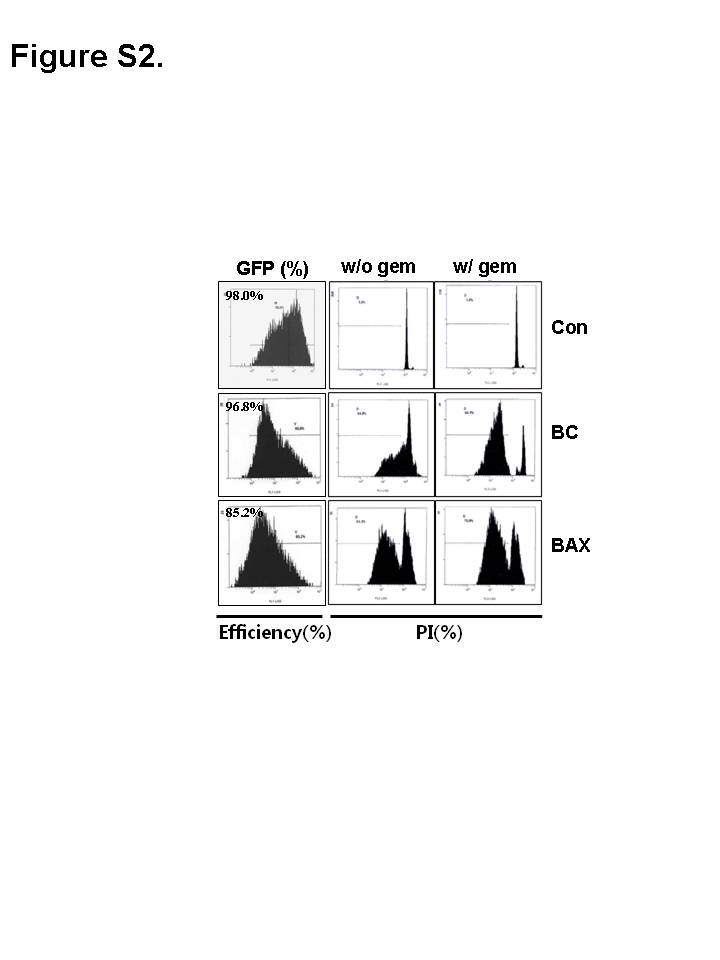

Supplement: Additional file 2 — Figure S2. BC was better in sensitizing lung cancer cells to gemcitabine than Bax. A549 cells infected with BC or BAX adenovirus were maintained in the presence or absence of 40 ng/ml gemcitabine for 72 h. SubG1 fractions were quantified by PI staining and FACS. GFP (%) represented infection efficiency of adenovirus constructs. [file 1476-4598-10-98-S2.JPEG]

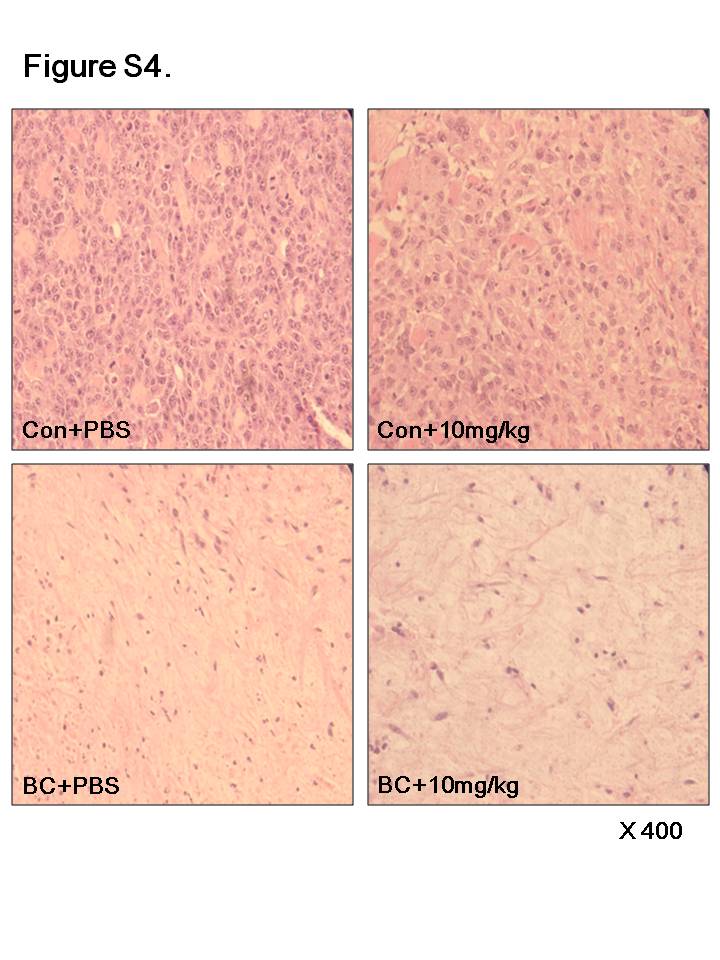

Supplement: Additional file 3 — Figure S3. Staining for Histological features. On day 30 after the last treatment, mice were sacrificed and tumors were extracted. The histogram shows the means ± SDs of tumor weights in each group (n = 5). Tumor sections were analyzed by hematoxylin and eosin staining for histologic features, such as, tumor necrosis and apoptosis. Numbers at the top of column indicates magnification. [file 1476-4598-10-98-S3.JPEG]

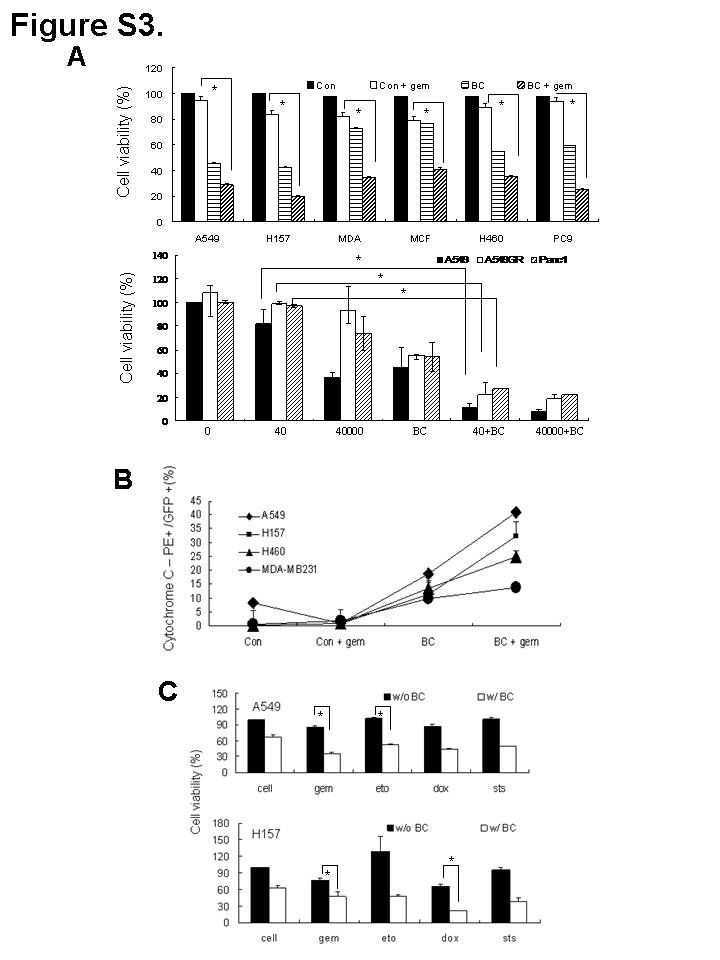

Supplement: Additional file 4 — Figure S4. BC synergistically compromised cell viability when administered with different chemotherapeutics to cancer cell lines. Human breast cancer cell lines, MDA-MB-231 (ATCC HTB-26, passage-8) and MCF7 (ATCC HTB-22, passage-13) and PANC-1(ATCC CRL-1469, passage-5) cells were verified by morphology, growth curve analysis and tested for Mycoplasma. All cell lines were obtained from and characterized by the American Type Culture Collection (ATCC). No further authentication was done by the authors. Etoposide, doxorubicin and staurosporine were purchased from LC Laboratories (Woburn, MA). A, MDA-MB-231 (MDA) and MCF7 (MCF) (human breast cancer cell lines), A549, H460, PC9 (human lung adenocarcinoma cell lines), and H157 (a human lung squamous cell carcinoma cell line) cells infected by BC or control adenovirus and Tet-off adenovirus, and then treated with 40 ng/ml gemcitabine for 72 h (upper panel). A549, gemcitabine-resistant A549 cells (A549GR), and PANC-1 (an intrinsic gemcitabine-resistant human pancreatic carcinoma cell line) were cells were infected with BC or control adenovirus and Tet-off adenovirus, and then treated with 0, 40, or 40000 ng/ml gemcitabine for 72 h (lower panel). Gemcitabine-resistant A549 cells (A549GR) were generated by exposure to increasing concentrations of gemcitabine from the first concentration of 40 ng/ml to final selection concentration of 4μg/ml. A549GR cells were passaged 4 to 5 times in the absence of drug about for 2 weeks, and maintained in the final selection concentration. Cell viabilities were measured using CCK-8 assays. Values are expressed as mean percentages of untreated cells in three independent experiments performed in triplicate; error bars represent SDs. B, A549, H157, H460 and MDA-MB-231 cells infected by BC or control adenovirus and Tet-off adenovirus treated with 40 ng/ml gemcitabine for 72 h. Cytochrome C release was analyzed by flow cytometry. Values are expressed as mean percentages of untreated cells in three [file 1476-4598-10-98-S4.JPEG]
